# The Effect of Heat Flux to the Fire-Technical and Chemical Properties of Spruce Wood (*Picea abies* L.)

**DOI:** 10.3390/ma14174989

**Published:** 2021-08-31

**Authors:** Martin Zachar, Iveta Čabalová, Danica Kačíková, Lucia Zacharová

**Affiliations:** 1 Department of Fire Protection, Faculty of Wood Sciences and Technology, Technical University in Zvolen, 960 01 Zvolen, Slovakia; zachar@tuzvo.sk (M.Z.); kacikova@tuzvo.sk (D.K.); 2 Department of Chemistry and Chemical Technologies, Faculty of Wood Sciences and Technology, Technical University in Zvolen, 960 01 Zvolen, Slovakia; 3 National Forest Centre, Forest Research Institute, 960 01 Zvolen, Slovakia; lucia.ambrusova@nlcsk.org

**Keywords:** spruce wood, heat flux, charring thickness, charring rate, chemical composition

## Abstract

The paper assesses the influence of the heat flux on spruce wood (*Picea abies* L.) behavior. The heat flux was performed at 15, 20, 25, and 30 kW·m^−2^. The fire-technical properties, such as the mass burning rate, charring thickness, charring rate, as well as the chemical composition (contents of the extractives, lignin, cellulose, holocellulose), of wood were determined. The highest burning rate of spruce wood of 0.32%·s^−1^ was reached at the heat flux of 30 kW·m^−2^. The charring rate ranged from 1.004 mm·min^−1^ (15 kW·m^−2^) to 2.016 mm·min^−1^ (30 kW·m^−2^). The proposed model of the charring process of spruce wood in time and appropriate thickness as a selected parameter is applicable in validation of the results of computer fire models in the design of fire protection of wooden buildings. The decrease in the holocellulose content mostly caused by the degradation of hemicelluloses was observed during thermal loading. The biggest decrease in hemicelluloses (24.94%) was recorded in samples loaded at 30 kW·m^−2^. The contents of cellulose increased due to the structural changes (carbonization and crosslinking), the content of lignin increased as well due to its higher thermal stability compared to saccharides, as well as the resulting lignin condensation.

## 1. Introduction

Wood and wood composites represent a main part of the fuel in many building fires [1]. The ability of materials to ignite when heated at elevated temperatures depends on many factors, such as the thermal properties [2,3,4], the chemical composition of materials [5,6,7], the ignition temperature [8,9,10], the heat flux, and the environment [1,11]. Under fire exposure, wood is degraded with heat transfer and weight loss [12,13]. During the combustion, the processes such as pyrolysis, ignition, radiation, and char formation occur in wood [14,15].

Charring rate is one of the most important fire properties of wood and wood products. It is one of the input data for calculation of fire resistance of wooden constructions according to STN EN 1995-1-2 (Eurocode 5) [16]. To enhance both the compression stability and fire resistance of wood, Chu et al. [17] used a new modification method; combined nitrogen–phosphorus (NP) fire retardant pre-impregnated with surface thermo-mechanical densification.

In addition, the charring rate of wood is a significant parameter in the determination of the fire cause, which is in line with NFPA 921: 2021 [18]. Technical standard (Eurocode 5) is the most reliable for charring depth analysis in assessment of the fire spread. To determine the burning time and the intensity of the fire, it defines the charring depth as a distance between the outer side of the surface of the original element and the position of the line between the charred layer and the rest of the cross-section. The standard considers the line of the charred layer of the wooden constructions as a place where the temperature reaches 300 °C. The charring rate of wood depends on the density, moisture content, heat flux, and oxygen concentration of the environment [19]. Fonseca and Barreira [20] measured the charred layer in five different places using thermocouples “K” and MGC Plus data acquisition system. The temperature was measured at different depths, i.e., 10, 20, 30, and 40 mm under the surface exposed to heat, and they found that the interface between thermal degraded and non-degraded wood is the border between the black and brown layer of the wood and is characterized by a temperature of 300 °C. According to White and Nordheim [21], the charred layer corresponds to a temperature of 288 °C. The results of the scientific papers by Findorák et al. [22] and Chen et al. [23] confirm that the rapid thermal decomposition of wood (in case of short-term exposure) begins at a temperature just below 300 °C. According to Babrauskas [24], the chairing rate has been the subject of interest of building safety experts, who wanted to determine the decrease in load-bearing capacity of wooden beams and columns in post-flashover. Standard test equipment to simulate flashover (test furnaces) was used to obtain these data. The test conditions were determined in line with ASTM E119–20 [25] or ISO 834–2 [26]. During his tests, the furnace was heated to a constant temperature. Spruce samples were put into the furnace and were exposed to the radiant environment of the furnace where the temperature ranged from 920 to 1070 °C.

Heating may cause changes in the main chemical components (cellulose, hemicelluloses, and lignin) and extractives of wood and their contents [6,27]. During thermal loading, hemicelluloses degrade first, resulting in the formation of various volatile compounds [28]. High temperatures cause also the degradation of lignin, resulting in the production of water, carbon dioxide, formic acid, acetic acid, and other substances that can be involved in condensation reactions [29]. Thermal degradation of the hemicelluloses starts at lower temperatures compared to lignin and cellulose [30]. According to the papers of Rantuch and Chrebet [31]; Gaan et al. [32], three steps of thermal decomposition of cellulose are indicated: the first stage at around 100 °C, which corresponds to the release of physically absorbed water; the second stage at around 360 °C (very rapid), corresponding to the dehydration and decarboxylation with production of combustible gasses; and the third stage (slow) at around 400 °C, which corresponds to the decomposition of the carbon formed in the second stage. Polleto [33] studied two wood species, *Pinus taeda* and *Eucalyptus grandis.* According to his results obtained from the thermogravimetric analysis, the water loss was observed at around 100 °C for both wood species. The thermal degradation occurred as a two-step process; in the first, the degradation of hemicelluloses took place at around 300 °C. The main degradation of cellulose occurs at around 350 °C for both species.

For an assessment of all the factors that influence the possibility of fire initiation, it is very important to know the fire-technical properties and the chemical composition of the wood. This paper deals with the influence of the heat flux (15, 20, 25, and 30 kW·m^−2^) on spruce wood behavior. Spruce wood is the most common coniferous tree in Slovakia and is very often used in construction. From the fire-technical properties, the mass burning and charring rate and chemical composition (lignin, cellulose, hemicelluloses and extractives) of spruce wood were evaluated. Based on the determined temperatures at individual depths of samples and using statistical methods of least squares, a model of the course of charring in the given time and depth was created.

Fire-technical properties, such as the mass burning rate, charring rate, and chemical composition (lignin, cellulose, hemicelluloses and extractives), of spruce wood were evaluated. Based on the determined temperatures at individual depths of 10, 20, 30, and 40 mm under the surface of test samples loaded with a ceramic infrared heater with a heat flux of 15, 20, 25, and 30 kW·m^−2^ and using statistical methods of least squares, a model of the course of charring in the given time and depth was created. This method has not been published in any original scientific paper yet. The paper will be useful to determine the fire investigation.

## 2. Materials and Methods

### 2.1. Materials

Samples for experimental work were made from spruce (*Picea abies* (L.) H. Karst.). A 120-year old tree was cut in location of Zvolen (altitude 800 m above sea level) in summer 2020. The dimension of the trunk was 420 mm and the moisture content was 41%. Subsequently, the trunk was cut using a band saw to a thickness of 55 mm and dried in a dryer to 15% moisture content. Test samples with diameters of 40 × 50 × 50 mm (transverse × radial × tangential direction) were dried to 0% moisture content, which was maintained until testing. The density of test samples was 443.61 ± 12 kg·m^−3^. Elementary analysis was conducted; its results are summarized in Table 1. Elementary analysis was carried out as follows: carbon (C) was determined according to STN ISO 10694 [34] using elementary analysis with thermally conductive analysis; nitrogen (N) according to ISO 13878 [35], using elementary analysis with thermally conductive analysis; sulfur (S) according to ISO 15178 [36], using elementary analysis with thermally conductive analysis; phosphor (P), calcium (Ca), magnesium (Mg), and potassium (K) according to ISO 11885 [37], using atomic emission spectrometry with inductively coupled plasma.

### 2.2. Methods

During the experiment, proposed measurement apparatus, in line with the utility model application No. 50121-2020, registered by the Industrial Property Office of the Slovak Republic, was used. The apparatus consists of a ceramic infrared heater (type F.T.E. with power of 1000 W, Ceramicx Ltd., Gortnagrough, Ireland), accurate digital scales (Radwag PS 3500.R2, RADWAG Balances and Scales, Radom, Poland), a control device (METREL HSN0203, Metrel d.d. Horjul, Slovenia), temperature measuring devices (Data logger ALMEMO^®^ 710, Ahlborn Mess- und Regelungstechnik GmbH, Holzkirchen, Germany), type “K” thermocouples with a thickness of 0.5 mm (Omega Engineering Inc., Norwalk, CT, USA), and an initiation burner (propane burner CLASIC CZ, Ltd., Řevnice, Czech Republic).

#### 2.2.1. The Mass Burning Rate

The reaction to fire tests were determined according to the EN ISO 11925–2 standard [38]. The mass burning rate was measured with apparatus described above. The sample was placed into the holder at a distance of 30 mm from the ceramic infrared heater, for a specific time of 1920 s, and the weight change was recorded every 10 s. The heat flux of the infrared heater was 15, 20, 25, and 30 kW·m^−2^. For the purpose of thermal loading with ceramic infrared heater, the samples were divided into 10 series of 5 samples for each value of the heat flux.

To determine the burning rate in the specified time interval, the absolute burning rate ʋ was calculated according to Equation (1):

ϑ = (δ(τ) − δ(τ + Δτ))/Δτ(1)

where:

- ϑ—absolute burning rate (%·s ^−1^);
- δ (τ)—sample weight in time (τ) (%);
- δ (τ + Δτ)—sample weight in time (τ + ∆τ) (%);
- Δ τ—time interval in which the weight values are recorded (s).

#### 2.2.2. Determination of the Charring Rate of Samples

To determine the course of temperatures in spruce samples, it was necessary to place thermocouples into them. Thermocouples were placed in test samples at a distance of 10 (T1), 20 (T2), 30 (T3), and 40 (T4) mm under the surface and were loaded with a heat flux of 15, 20, 25, and 30 kW·m^−2^.

Based on the recorded course of temperatures in the samples, we could determine the course of degradation of spruce wood without the need to manually measure the charring thickness. As it is stated in NFPA 921:2017 [18], the charring rate of wood depends on the amount of wood and a large number of variables (characteristics of wood) and fire conditions. To determine the burning time and fire intensity, STN EN 1995—1—2 (Eurocode 5) [16] can be applied, stating that the line of charred layer of wooden constructions is considered as a place where the temperature reaches 300 °C. Reaching a temperature of 300 °C in test samples (at depths of 10, 20, 30, and 40 mm) was also decisive for our measurements to determine the depth of charring.

#### 2.2.3. Chemical Composition of Wood

Samples were disintegrated into sawdust, and fractions from 0.5 mm to 1.0 mm in size were used for the chemical analyses. According to ASTM D1107-21 [39], the extractives content was determined in a Soxhlet apparatus with a mixture of ethanol and toluene (2:1). The lignin content was determined according to Sluiter et al. [40], the cellulose according to the method by Seifert [41], and the holocellulose according to the method by Wise et al. [42]. Hemicelluloses were calculated as a difference between the holocellulose and cellulose content. Measurements were performed on four replicates per sample. The results were presented as oven-dry wood percentages.

## 3. Results and Discussion

### 3.1. Burning Rate of Test Samples

**Figure 1:**
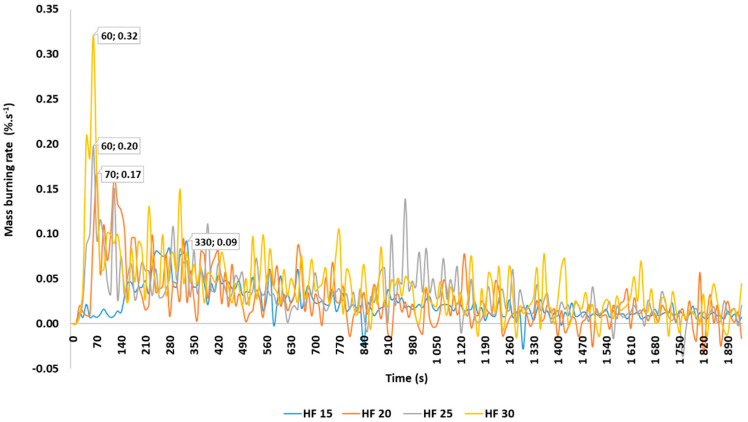
Absolute burning rate of the spruce wood samples.

The results of mass burning rate are shown in Figure 1, where the maximum burning rate values, as well as time of reaching these maximum burning rate values, are referred to depending on the heat flux of 15, 20, 25, and 30 kW·m^−2^.

Burning rate of spruce wood samples is calculated based on weight losses in time interval up to 1920 s.

From the fire protection point of view, the time taken for the tested material to reach the given value of the flash point plays an important role. The results of our measurements show that, with increasing heat flux, the time to initiation of spruce samples decreases. At the thermal loading with a heat flux of 15 kW·m^−2^, the time to initiation was significantly higher (261.5 s), with a maximum burning rate of 0.09%·s^−1^. At the thermal loading with a heat flux of 20 kW·m^−2^, the time to initiation was 47.5 s and maximum burning rate was 0.17%·s^−1^. At the thermal loading with a heat flux of 25 kW·m^−2^, the time to initiation was 22.0 s and the maximum burning rate was 0.17%·s^−1^. At the thermal loading with a heat flux of 30 kW·m^−2^, the time to initiation was 11.25 s and the maximum burning rate was 0.32%·s^−1^. The time of reaching the maximum burning rate is changes significantly, depending on the heat flux. With increasing values of the heat flux, the interval for reaching the maximum burning rate of spruce samples decreases. According to the paper by Zachar et al. [43], in determining the time to initiation, in line with STN ISO 871 [44], for spruce wood samples prepared from root, branch, and trunk, we could observe a greater variability of the results. Values of ignition times ranged from 420 s for branch, 485 s for root, and up to 560 s for trunk. Delichatsios et al. [45], in testing spruce wood, reached an ignition temperature of approximately 478 °C and an initiation time of 266 s, which is comparable to our results. Hagen et al. [46], in testing spruce samples, reached an ignition temperature of approximately 488 °C and an initiation time of 253 s. Makovická-Osvaldová et al. [47] assessed the initiation time of different tree species with dimensions of 9 × 9 × 1 cm, loaded with a heat flux of 35 kW·m^−2^, using conical calorimeter. In the case of spruce wood, they stated that the initiation time ranges from 30 to 54 s, which is comparable to our results. The burning rate was also measured by Gaff et al. [48], who reached a comparable burning rate of untreated teak wood of 0.18%·s^−1^ in an initiation time of 174 s; in the case of thermally treated samples, values of the maximum burning rate were higher, ranging from 0.23 to 0.33%·s^−1^. Kačíková and Makovická [49] compared the burning rate of coniferous species, finding that the lowest rate of 0.09%·s^−1^ was reached for spruce wood. Makovická-Osvaldová et al. [50] determined the burning rate of spruce wood loaded with propane burner in a time interval from 0.08 to 0.095%·s^−1^, which is comparable to our results.

### 3.2. Charring Rate of Test Samples

The charring rate was determined based on time measurement until a temperature of 300 °C was reached (Table 2).

An average temperature of 300 °C reached on individual thermocouples T1, T2, and T3 loaded with a heat flux of 15, 20, 25, and 30 kW·m^−2^ corresponds to the respective charring depth and, subsequently, to charring rate. The lowest charring rates were reached under a thermal load of 15 kW·m^−2^, i.e., 1.188 mm·min^−1^ in a time of 505 s (T1) and 1.004 mm·min^−1^ (T2) in a time of 1195 s. On other thermocouples (T3 and T4) at a thermal loading of 15 kW·m^−2^, a temperature of 300 °C was not reached within 1920 s (end of thermal loading of samples); therefore, it was not possible to determine the charring depth and rate. At a thermal loading of 20 kW·m^−2^, a temperature of 300 °C was reached in a time of 391 s (T1), and the charring rate was 1.535 mm·min^−1^; on thermocouple T2, in a time of 811 s, the charring rate was 1.479 mm·min^−1^. On other thermocouples (T3 and T4), at a thermal loading of 20 kW·m^−2^, a temperature of 300 °C was not reached within 1920 s. At a thermal loading of 25 kW·m^−2^, a temperature of 300 °C was reached in a time of 298 s (T1), and the charring rate was 2.013 mm·min^−1^; on thermocouple T2, the charring rate was 1.653 mm·min^−1^, and on thermocouple T3, it was 1.424 mm·min^−1^, which indicates that the charring rate decreased towards the center of the sample. On thermocouple T4, at a thermal loading of 25 kW·m^−2^, a temperature of 300 °C was not reached within 1920 s. At the highest thermal loading of 30 kW·m^−2^, a temperature of 300 °C was reached in a time of 314 s (T1), and the charring rate was 2.911 mm·min^−1^; on thermocouple T2, the charring rate of 2.016 mm·min^−1^ was reached in a time of 596 s; on thermocouple T3, the charring rate of 1.842 mm·min^−1^ was reached in a time of 977 s (which are the shortest times of charring). On thermocouple T4, at a thermal loading of 30 kW·m^−2^, a temperature of 300 °C was not reached within 1920 s; therefore, it was not possible to determine the charring depth and rate.

According to NFPA 921: 2017 [18], the charring rate of wood under laboratory conditions and exposure to a heat source from one side is determined from 0.17 mm·min^−1^ to 4.23 mm·min^−1^. Lipinskas and Mačiulaitis [51] investigated the charring of fir samples treated with various kinds of retardant using different test equipment. Using a heating chamber, they determined the charring rate of selected coniferous tree species, ranging from 0.6 to 1.1 mm·min^−1^.

**Figure 2:**
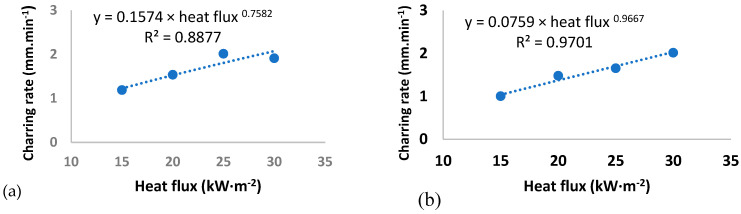

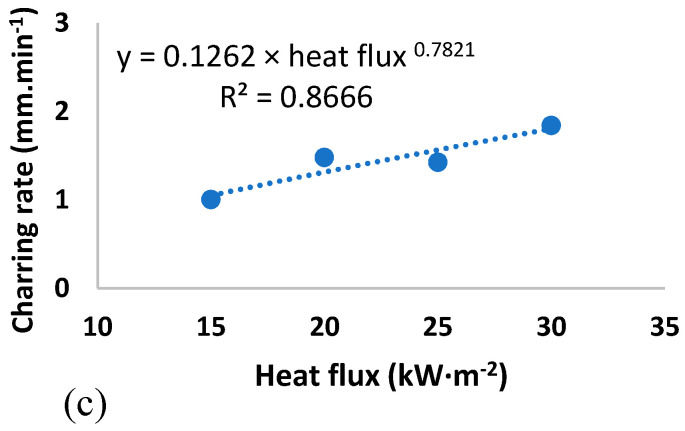
Average charring rate in time interval: (**a**) from 0 to 600 s; (**b**) from 0 to 900 s; (**c**) from 0 to 1920 s.

The relationship between average charring rate of spruce wood and the heat flux density (in time intervals from 0 to 600 s; from 0 to 900 s; and from 0 to 1920 s) is shown in Figure 2.

The results show that the charring rate increases with increasing heat flux. In the time interval from 0 to 600 s, the average charring rate ranged from 1.18 mm·min^−1^ (at a heat flux of 15 kW·m^−2^) to 2.01 mm·min^−1^ (at a heat flux of 30 kW·m^−2^). In the time interval from 0 to 900 s, the average charring rate ranged from 1.00 mm·min^−1^ (at a heat flux of 15 kW·m^−2^) to 2.02 mm·min^−1^ (at a heat flux of 30 kW·m^−2^). In the time interval from 0 to 1920 s, the average charring rate ranged from 1.00 mm·min^−1^ (at a heat flux of 15 kW·m^−2^) to 1.84 mm·min^−1^ (at a heat flux of 30 kW·m^−2^). Martinka et al. [52] described a significant linear relationship between the charring depth and the ratio of mass loss to density. The average charring rate decreases with increasing time, and increases with increasing heat flux. He stated that the average charring rate of spruce wood ranged from 0.73 mm·min^− 1^ (at a heat flux of 20 kW·m^−2^) to 1.2 mm·min^−1^ (at a heat flux of 50 kW·m^−2^). The reason for the decreasing charring rate is mainly that the charred layer formed on the surface of the sample reduces the overheating and thermal decomposition of non-degraded wood under the charred layer. Similar results were also reached by other authors, e.g., Zhang [53] and Lizhong et al. [54], who tested various tree species at a heat flux of 15, 25, 35, and 50 kW·m^−2^. Their conclusions show that the charring rate of wood exposed to a constant external heat flux can be considered as a linear function of time, but at a higher levels of heat flux, its behavior can be nonlinear, with longer intervals needed to reach a given charring depth. The rate of charring is directly proportional to the ratio of exposure to external heat flux and density. Based on the determined course of temperatures at individual depths (10, 20, 30, and 40 mm) under the surface of test samples of spruce wood loaded with a ceramic infrared heater with a heat flux of 15, 20, 25, and 30 kW·m^−2^, and using statistical method of least squares, we can develop a model of the course of charring in time and appropriate thickness. Reaching a temperature of 300 °C was considered critical, or marginal.

**Figure 3:**
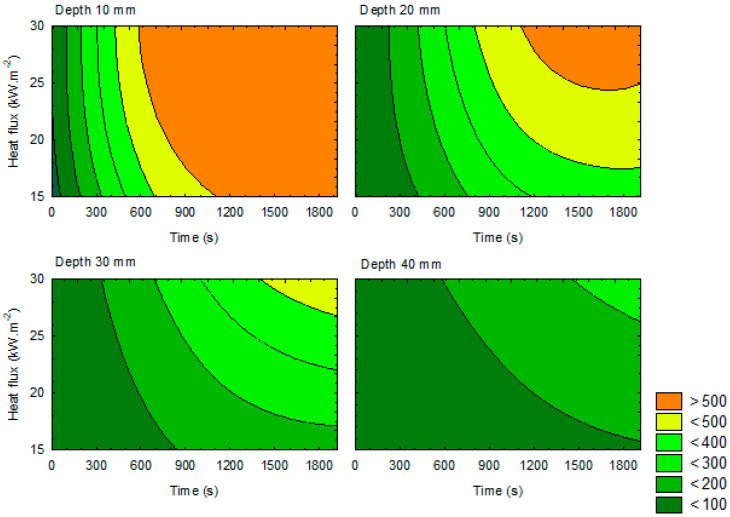
Model of the course of charred layer formation in time for respective depth.

Figure 3 shows a model of the course of charred layer formation depending on heat flux loading (15, 20, 25, and 30 kW·m^−2^) at individual depths (up to 10, 20, 30, and 40 mm) under the surface of test samples of spruce wood loaded with ceramic infrared heater in a time interval from 0 to 1920 s.

The marginal temperature needed to reach a charred layer is shown in light green. It is a temperature field reaching a temperature of 300 °C in the entire time interval (from 0 to 1920 s) of thermal loading with a ceramic infrared heater. The course of charred layer formation up to a depth of 10 mm under the surface is shown upper left. The rate and extend of charred layer formation clearly indicates the dependance on the heat flux and time of loading. At a heat flux of 15 kW·m^−2^, the charred layer starts to form in a time of 200 s from the beginning of the loading; at a heat flux of 20 kW·m^−2^, it forms in a time of 120 s; at a heat flux of 25 kW·m^−2^, it forms in a time of 100 s; and at a heat flux of 30 kW·m^−2^, it forms in a time of 95 s. Based on the measurement of temperature courses in test samples on thermocouple T1, different times of reaching the critical temperature of 300 °C were recorded. The measurements show that, for charred layer formation up to a thickness of 10 mm under the surface, the critical heat flux at a thermal load of 15 kW·m^−2^ occurs in 505 s, at a thermal load of 20 kW·m^−2^ occurs in 395 s, at a thermal load of 25 kW·m^−2^ occurs in 300 s, and at a thermal load of 30 kW·m^−2^ occurs in 315 s, i.e., in initiation phase of the fire. The course of the charred layer formation at a depth from 10 to 20 mm under the surface of test samples is shown in Figure 3 (upper right). The rate and extend of the charred layer formation also indicates the dependance on the heat flux and time of loading. At a heat flux of 15 kW·m^−2^, the charred layer starts to form in a time of 760 s from the beginning of loading, at a heat flux of 20 kW·m^−2^ in 550 s, at a heat flux of 25 kW·m^−2^ in 460 s, and at a heat flux of 30 kW·m^−2^ in 415 s. It means that, for the charred layer formation at a depth from 10 to 20 mm under the surface of the test sample, the critical heat flux is 30 kW·m^−2^, reached in a time of 415 s, i.e., in the third quarter of initiation phase of the fire (up to 600 s from the beginning of loading). The course of the charred layer formation at a depth from 20 to 30 mm under the surface of test samples is shown in Figure 3 (bottom left). The rate and extent of the charred layer formation indicate the dependance on the heat flux and time of loading. At a heat flux of 15 kW·m^−2^, the charred layer was not formed within 1920 s. At a heat flux of 15 kW·m^−2^, the critical temperature of 300 °C (recorded on thermocouple T3) was not reached during the entire time interval of thermal loading. At a heat flux of 20 kW·m^−2^, the charred layer starts to form in a time of 1 180 s, at a heat flux of 25 kW·m^−2^ in a time of 880 s, and at a heat flux of 30 kW·m^−2^ in a time of 715 s. The given times indicates that, for the formation of the charred layer at a depth from 20 to 30 mm under the surface of the test samples, the critical heat flux is 30 kW·m^−2^ in a time of 715 s, i.e., after the initiation phase of the fire (up to 600 s from the beginning of loading), which is partly in conflict with our measurements, because, at a loading of 30 kW·m^−2^, the critical temperature of 300.9 °C (recorded on thermocouple T3) was reached in a time of 980 s from the beginning of thermal loading.

The course of charred layer formation at a depth from 30 to 40 mm under the surface of test samples is shown in Figure 3 (bottom right). At a heat flux of 15, 20, and 25 kW·m^− 2^, the charred layer was not formed within 1920 s. According to our measurement of the temperature course at a loading of 15, 20, 25 and 30 kW·m^−2^, the critical temperature of 300 °C (recorded on thermocouple T4) was not reached during the entire time interval of thermal loading. According to the illustration in Figure 3, the formation of charred layer at a depth of 40 mm under the surface occurs at a thermal loading of 30 kW·m^−2^ in a time of 1490 s, i.e., in the phase of fully developed fire.

### 3.3. Changes in the Chemical Composition

The effect of heat flux loading on chemical composition of wood is shown in Table 3.

The data show that, with increasing heat flux value, both the relative content of lignin and cellulose increases. Wang et al. [55] assessed the effect of thermal modification on the chemical properties of Masson pine (*Pinus massoniana*) wood. Based on their results, the relative content of lignin and cellulose increases with increasing temperature and time of thermal loading. The highest values of the abovementioned chemical components were obtained in samples loaded by a heat flux of 30 kW·m^−2^. The increase in lignin content in the thermally loaded wood indicated its higher thermal stability compared to saccharides, as well as the resulting lignin condensation [56]. The increase in the cellulose content is described as its structural changes (carbonization and crosslinking) [6].

Compared to the original samples, the lowest values of hemicelluloses (decrease 24.94%) were measured in the samples thermally loaded at higher heat flux. Wood degradation starts with deacetylation of hemicelluloses, and is followed by the depolymerization of polysaccharides, which is catalyzed by the released acetic acid [57]. The lower thermal stability of hemicelluloses than of the cellulose is visible in the C/H ratio. It is very reliable indicator for the rate assessment of the lower stability of hemicelluloses towards cellulose [58]. Compared to the original samples, the C/H ratio of samples loaded at 30 kW·m^−2^ is the highest (52%); the content of extractives increased in samples loaded at 20 and 30 kW·m^−2^ and decreased in samples loaded at 15 and 25 kW·m^−2^. Several authors [59,60] described a decrease and others [56,61,62] an increase in extractives content in thermally loaded wood. The increase in extractives is explained by products of thermal degradation of the lignin and polysaccharides macromolecule [63].

## 4. Conclusions

Fire-technical properties, such as the mass burning rate and charring rate, and chemical composition (lignin, cellulose, hemicelluloses and extractives), of spruce wood were evaluated. Based on the determined temperatures at individual depths of 10, 20, 30, and 40 mm under the surface of test samples loaded with a ceramic infrared heater with a heat flux of 15, 20, 25, and 30 kW·m^−2^, and using statistical methods of least squares, a model of the course of charring in the given time and depth was created. This method has not been published in any original scientific paper yet.

Based on a comparison of average burning rates (at a heat flux of 15, 20, 25, and 30 kW·m^−2^) reached at thermal loading of samples using a ceramic infrared heater, and using our proposed test method, it can be stated that, with increasing heat flux, initiation time and time to reach the maximum burning rate of spruce wood—ranging from 0.09%·s^−1^ (15 kW·m^−2^) to 0.32%·s^−1^ (30 kW·m^−2^)—decrease.

Based on the determination of temperature course using thermocouples (type “K”) placed at a distance of 10, 20, 30, and 40 mm under the surface of samples loaded with a ceramic infrared heater, we could determine the burning rate in a time interval from 0 s to 1920 s, which corresponds to the first three phases of the fire without the need of manual measurement. The burning rate ranges from 1.004 mm·min^−1^ at a heat flux of 15 kW·m^−2^ to 2.016 mm·min^−1^ at a heat flux of 30 kW·m^−2^.

Based on the course of temperatures, and using the statistical method of least squares, a model of the course of charring in time a respective thickness was created. The temperature of 300 °C is considered as marginal temperature of charring, which is a benefit over the methods applied in the past.

The proposed model of the course of charring will serve for validation of computer fire models, and subsequently, the results can be used in the design and solutions for fire protection of wooden constructions.

The relative content of both lignin and cellulose increases, and hemicelluloses decreases, with higher values of heat flux loading. The lower stability of hemicelluloses towards cellulose was demonstrated via the cellulose/hemicelluloses ratio (C/H), whereas the ratio increases with higher values of heat flux loading.

The proposed model of the course of charring will serve for validation of computer fire models, and subsequently, the results can be used in the design and solutions for fire protection of wooden constructions and as an input data for computer modelling in simulation software Ansys.

## Figures and Tables

**Table 1:** Elementary analysis of spruce wood used for the experiment.

Element	C (g·kg^−2^)	N (g·kg^−2^)	S (mg·kg^−2^)	P (g·kg^−2^)	Ca (g·kg^−2^)	Mg (g·kg^−2^)	K (g·kg^−2^)
Spruce wood	489	1.32	299	0.151	0.702	0.044	0.621

**Table 2:** Thickness and charring rate of test samples. The values are the mean ± the SD.

Heat Flux (kW·m^−2^)	Time of Thermal Loading (s)	Charring Thickness (mm)	Charring Rate (mm·min^−1^)
15	505 ± 5.50	10 ± 0.15	1.188
15	1195 ± 6.20	20 ± 0.05	1.004
15	-	-	-
20	391 ± 1.40	10 ± 0.13	1.535
20	811 ± 4.80	20 ± 0.72	1.479
20	-	-	-
25	298 ± 3.10	10 ± 0.18	2.013
25	726 ± 6.60	20 ± 0.25	1.653
25	1364 ± 7.10	30 ± 0.51	1.424
30	314 ± 2.50	10 ± 0.09	1.911
30	596 ± 3.30	20 ± 0.35	2.016
30	977 ± 5.00	30 ± 0.46	1.842

**Table 3:** Chemical composition of the spruce wood before and after thermal loading (relative values).

Heat Flux (kW·m^−2^)	Extractives (%)	Lignin (%)	Cellulose (%)	Holocellulose (%)	Hemicelluloses (%)	C/H * Ratio
Original	1.40 ± 0.00	25.48 ± 0.08	41.23 ± 0.03	73.11 ± 0.08	31.88 ± 0.11	1.29
15	1.09 ± 0.00	26.04 ± 0.04	43.24 ± 0.08	72.88 ± 0.04	29.64 ± 0.12	1.46
20	1.51 ± 0.01	26.26 ± 0.01	43.14 ± 0.00	72.23 ± 0.01	29.09 ± 0.01	1.48
25	1.19 ± 0.00	26.35 ± 0.33	45.90 ± 0.42	72.46 ± 0.33	26.56 ± 0.09	1.73
30	1.54 ± 0.01	27.60 ± 0.23	46.92 ± 0.17	70.85 ± 0.22	23.93 ± 0.05	1.96

* C/H ratio—cellulose/hemicelluloses ratio. The values are the mean ± the SD.
